# Formulation and Characterization of Hesperidin-Loaded Transethosomal Gel for Dermal Delivery to Enhance Antibacterial Activity: Comprehension of In Vitro, Ex Vivo, and Dermatokinetic Analysis

**DOI:** 10.3390/gels9100791

**Published:** 2023-10-01

**Authors:** Perwez Alam, Mohd Imran, Samreen Jahan, Ali Akhtar, Zafrul Hasan

**Affiliations:** 1 Department of Pharmacognosy, College of Pharmacy, King Saud University, P.O. Box 2457, Riyadh 11451, Saudi Arabia; aakhtar@ksu.edu.sa; 2 Department of Pharmacognosy, School of Pharmaceutical Education and Research, Jamia Hamdard, New Delhi 110062, India; imranidrisi00786@gmail.com; 3 Department of Pharmaceutics, School of Pharmaceutical Education and Research, Jamia Hamdard, New Delhi 110062, India; samreenj1996@gmail.com; 4 Department of Medical Surgical Nursing, College of Nursing, King Saud University, Riyadh 11451, Saudi Arabia; zhasan@ksu.edu.sa

**Keywords:** hesperidin, transethosome, positive bacteria, negative bacteria, antimicrobial activity, bacterial skin infection

## Abstract

In this study, hesperidin was loaded into a transethosome and was developed employing the rotary evaporator method. The formulation was optimized using the Box–Behnken design (BBD). The optimized HSD-TE formulation has a spherical shape, vesicle size, polydispersity index, entrapment efficiency, and zeta potential within the range of 178.98 nm; the PDI was 0.259 with a zeta potential of −31.14 mV and % EE of 89.51%, respectively. The in vitro drug release shows that HSD-TE exhibited the release of 81.124 ± 3.45% in comparison to HSD suspension. The ex vivo skin permeation showed a 2-fold increase in HSD-TE gel permeation. The antioxidant activity of HSD-TE was found to be 79.20 ± 1.77% higher than that of the HSD solution. The formulation showed 2-fold deeper HSD-TE penetration across excised rat skin membranes in confocal laser microscopy scanning, indicating promising in vivo prospects. In a dermatokinetic study, HSD-TE gel was compared to HSD conventional gel where TE significantly boosted HSD transport in the epidermis and dermal layers. The formulation showed greater efficacy than free HSD in the inhibition of microbial growth, as evidenced by antibacterial activity on the Gram-negative and positive bacteria. These investigations found that the HSD-TE formulation could enhance the topical application in the management of cutaneous bacterial infections.

## 1. Introduction

Skin diseases are prevalent and significantly affect the quality of life of individuals [1]. According to the International Classification of Diseases, there are over 1000 skin or skin-related diseases categorized into 10 distinct categories [2]. Remarkably, skin and subcutaneous diseases were positioned at the 18th spot in the global ranking of disease burden measured in Disability-Adjusted Life Years (DALYs) according to the Global Burden of Disease in 2013. Additionally, skin diseases held the fourth highest position as a cause of disability on a global scale [3]. Bacterial skin infections constitute a significant proportion within the category of skin diseases. Nevertheless, despite the significant influence of bacterial skin diseases on the overall disease burden worldwide, there seems to be a noticeable absence of corresponding global attention [4]. 

Hesperidin (HSD) is categorized as a secondary metabolite of plants and represents a prominent bioflavonoid constituent inside citrus fruit. The flavanone compound, along with its aglycone derivative known as hesperetin (HST) can be found in significant proportions (1–2%) within sweet immature oranges (*Citrus sinensis*) (Stanisic et al., 2020) [5]. Hesperidin is recognized for its significant bioactivity and was found to possess antioxidant [6], anti-inflammatory [7], hypolipidemic [8], vasoprotective [9], and anti-anticancerogenic [10] properties. Pharmacological investigations demonstrated that HSD exhibits pharmacological advantages, including the ability to lower blood pressure, combat bacterial and viral infections, and enhance immune function [11]. Consequently, HSD holds promising potential for application in the realms of functional foods and medicine.

Nevertheless, the bioavailability of HSD is low, including low water solubility [12], leading to restricted transmembrane permeability [13], thereby significantly restricting its potential for clinical utilization [14]. There was a recent increase in research focused on the utilization of nanoencapsulation techniques to enhance the bioavailability of drugs and phytocompounds with low bioavailability [15]. The objective of enhancing drug effectiveness is a primary focus in the investigation and advancement of innovative drugs [16]. 

The use of drug delivery systems utilizing phospholipids was recognized as a viable strategy for the percutaneous distribution of active compounds, owing to the innate biocompatibility and biodegradability of phospholipids. However, the outermost layer of skin’s resistance can frequently stop drugs from penetrating the skin. To overcome the stratum corneum barrier and allow drugs to be absorbed via the skin, researchers have explored several different approaches [17]. 

The utilization of ethosomal carriers presents a potentially advantageous approach for the delivery of HSD and other bioactive compounds. The fusion of ethosomes with bacterial cell membranes was observed to enhance the efficacy of antimicrobial drugs, irrespective of the drugs’ solubility [18,19]. Ethosomes are commonly recognized as modified versions of liposomes, which consist of phospholipids and a relatively elevated concentration of ethanol and water. These structures were developed to facilitate the delivery of drugs that exhibit both hydrophilic and lipophilic properties [20]. Ethanol demonstrates the capability to enhance the penetration of ethosomes through pores, facilitating their deeper permeation into the deeper layers. Consequently, this enables a more efficient release of drugs into deeper layers compared to conventional nanocarriers [21]. Transethosomes (TE) are lipid-based vesicles that incorporate both edge activators and ethanol, thereby synergistically harnessing the advantages of deformable liposomes and ethosomes [22,23]. 

The integration of nano-formulations into gel systems was found to enhance drug permeability and residence time, thereby improving therapeutic efficacy. Gelling agents and humectants are key factors in the preparation process that significantly impact the physical quality and stability of gel formulations. The formation of a gelling agent contributes significantly to the establishment of a structural system, which is a crucial determinant in the overall composition of the gel. The inclusion of humectants in the gel preparation serves to uphold its stability by facilitating moisture absorption and minimizing water evaporation [24]. Carbopol is a synthetic polymer derived from acrylic acid and offers effective viscosity control [25]. The consideration of carbopol 934 as a gelling agent stems from its notable attributes, such as its exceptional stability, resistance to microbial degradation, and extensive utilization in both the pharmaceutical and cosmetic sectors [24].

The objective of this study was to examine the potential antibacterial properties of HSD against *Staphylococcus aureus* and *Escherichia coli*, both Gram-negative and positive bacteria. This bacterium is widely recognized as the predominant causative agent of skin infections [26,27]. The aim of this study was to develop a transethosomal gel formulation containing HSD, with the goal of enhancing the topical delivery of HSD. The transethosome containing HSD was formulated and optimized via the utilization of the Box–Behnken design (BBD). The transethosomes containing optimized HSD were integrated into a carbopol 934-based system. Finally, the acquired preparation was subsequently assessed for various physical properties, such as particle size, zeta potential, transmission electron microscopy, in vitro drug release, ex vivo permeation, confocal laser microscopy scanning, dermatokinetic, and antimicrobial efficacy. Based on the available literature, no research was conducted to encapsulate hesperidin in transethosome carriers for antibacterial activity. 

## 2. Results and Discussion

### 2.1. Preparation of HSD-Loaded Transethosomes 

Hesperidin-loaded transethosomes were successfully prepared by utilizing the rotary evaporator method. 

### 2.2. Optimization of HSD-Loaded Transethosome 

**Figure 1:**
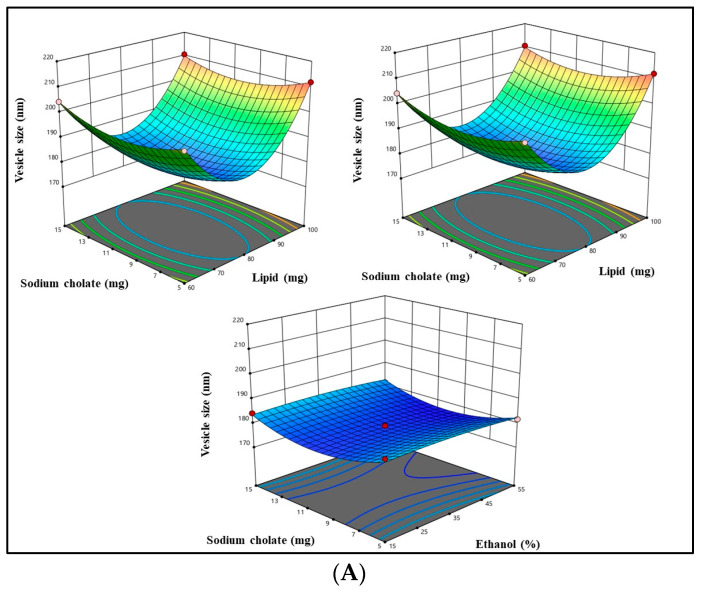

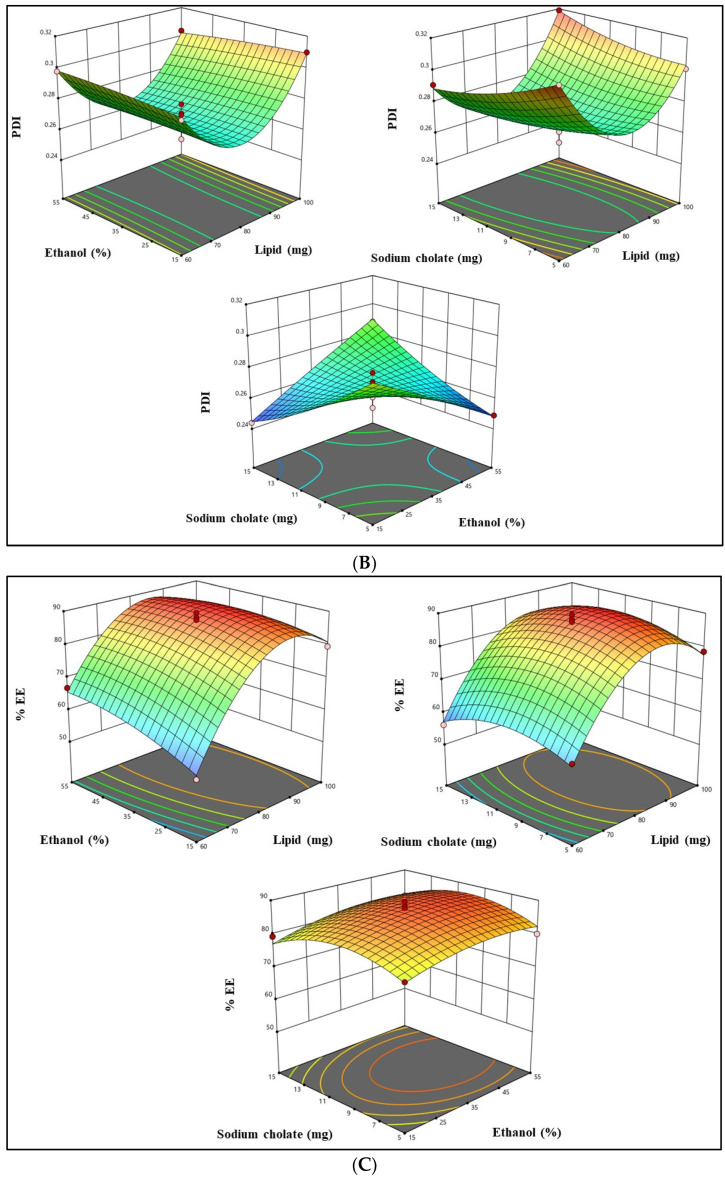
(**A**) The figure displays a three-dimensional surface plot depicting the correlation between independent variables and vesicle size. (**B**) A three-dimensional surface plot depicting the correlation between independent variables and PDI. (**C**) A three-dimensional surface plot depicting the correlation between independent variables and % entrapment efficiency.

The current study utilized a Box–Behnken design to assess the interactions and effects of formulation-independent parameters at varying levels on three significant quality attributes: vesicle size, PDI, and % encapsulation efficiency (Y_1_, Y_2_, and Y_3_). The experimental results of the Box–Behnken design are summarized in Table 1. The relationship between various independent variables such as phospholipid 90G (mg) (A), Ethanol (%), and sodium cholate (mg) (C), and dependent variables such as vesicle size (nm), PDI, and % EE is illustrated in the 3D graph presented in Figure 1A–C. The quadratic model was found to be the most appropriate fit for all the responses under consideration. The statistical significance of the regression coefficients for the input factors was assessed using the corresponding *p*-value, which was calculated via analysis of variance (ANOVA) and is reported in Table 2. The utilization of *p*-values served to assess the statistical significance of each coefficient, thereby facilitating an examination of the underlying patterns of mutual interactions among the chosen process parameters. As demonstrated in Table 2, a *p*-value of 0.05 or less was considered to indicate that the factor had a statistically significant impact. The model’s adequacy was assessed using the coefficient of determination (R^2^).

#### 2.2.1. Response (Y_1_): Impact of Independent Variable on Vesicle Size

According to the polynomial equation, sodium cholate and ethanol had a negative impact on vesicle size, while phospholipid 90G showed a beneficial effect.

Vesicle size = +178.53 + 3.71A − 0.6525B − 0.0712C − 0.5475AB − 0.9050AC + 0.0925BC + 23.19A^2^ − 0.9625B^2^ + 5.53C^2^

The vesicle size ranges from around 178.14 to 211.98 nm in the developed formulation, as shown in Table 1. An increase in the concentration of phospholipid 90G was found to be associated with an observed increase in the size of transethosome vesicles. Increased concentrations and enhanced availability of lipids result in greater entrapment of the drug, leading to an increase in vesicle size. The lowest vesicle size can be attained with a reasonable phospholipid concentration since the optimal vesicle size must have the ideal lipid content. The findings of our study, which are in line with other research, demonstrate that the size of the vesicle increases as the concentration of lipids in the formulation increases [28,29,30]. Beyond a certain threshold concentration, the cellular membrane’s bilayer structure may be disrupted, leading to a decrease in vesicle size correlated with an increase in sodium cholate and ethanol concentration [31]. 

#### 2.2.2. Response (Y_2_): Impact of Independent Variable on PDI

As per the polynomial Equation below, all the independent variables were found to be exhibiting a negative effect on PDI.

PDI = +0.990150 − 0.014886A − 0.0017B − 0.02053C − 6.2500AB + 0.000105AC + 0.000235BC + 0.00008A^2^ − 2.000B^2^ + 0.000168 C^2^

The PDI ranges from around 0.244 to 0.319 of the developed formulation, as shown in Table 1. Two reasons may be responsible for the observed decline in PDI value as the concentration of lipid and ethanol rises: a reduction in vesicle size at higher ethanol concentrations and an improvement in vesicle smoothness with increasing lipid content. These elements support a decrease in particle agglomeration, leading to an increase in homogeneity [32]. Lower PDI is caused by similar effects when the concentration of sodium cholate is raised from 05 to 15 mg. The results of our investigation are consistent with prior studies [31]. 

#### 2.2.3. Response (Y_3_): Impact of Independent Variable on %EE

According to the polynomial equation below, phospholipid 90G and ethanol were discovered to have a favorable impact on entrapment efficiency, whereas sodium cholate had a negative impact.

%EE = +87.4 + 9.72A + 2.10B − 0.5150C − 2.65AB + 0.5375AC + 0.2725BC − 14.45A^2^ − 2.40B^2^ − 5.03C^2^

The %EE ranges from around 56.19 to 89.51% of the developed formulation, as shown in Table 1. The observed increment in % EE as the concentration of phospholipid increases might be attributed to the increase in domain size of the bilayer ensuing from the formation of a higher quantity of TE vesicles. This, in turn, allows for a greater capacity for HSD entrapment within the TE vesicles [33]. The efficiency of the transethosomal system is significantly impacted by ethanol as well; raising the concentration of ethanol will enhance entrapment efficiency. Ethanol increases the solubility of the lipophilic and amphiphilic drug, increasing drug loading. This effect is applicable to molecules of varied lipophilicities. With ethanol concentrations ranging from 15% to 55%, it was discovered that this connection was linear. Because phospholipids can easily dissolve in ethanol, the transethosomal membrane will be more permeable at very high concentrations, which will result in a significant decrease in entrapment efficacy. For this reason, ethanol concentration should be optimized during the formulation process [34]. The decline in entrapment efficiency resulting from the elevation of sodium cholate concentration may be attributed to the phenomenon wherein sodium cholate, beyond a specific concentration, disrupts the structural integrity of the vesicular bilayered membrane. Consequently, this disruption causes the drug to be released from the vesicles, resulting in a loss of drug encapsulation [31].

### 2.3. Characterization of HSD-TE Formulation 

#### 2.3.1. Vesicle Size and PDI

**Figure 2:**
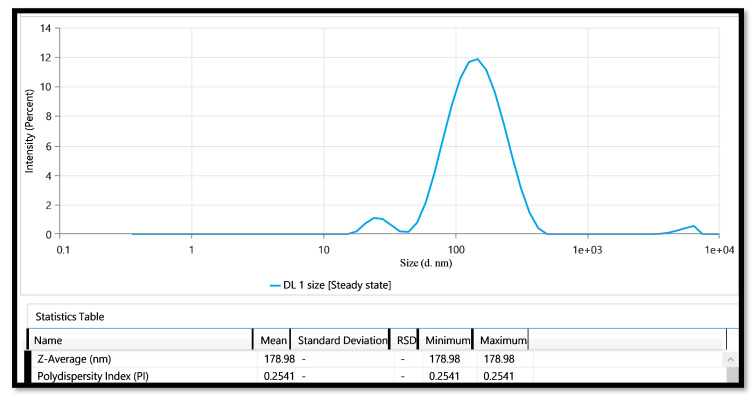
Vesicle size and PDI of Opt-HSD-loaded TE formulation.

The vesicle size of the optimized HSD-loaded transethosome (Opt-HSD-TE) was found to be 178.98 nm (Figure 2). The dimensions of the vesicles in the formulation are of the utmost significance in facilitating the skin absorption of the drug. Additionally, according to some studies, hair follicles act as long-term drug storage sites, holding drugs that are given topically for up to ten times longer than the SC’s reservoir. The reduced size of nanovesicles enables enhanced skin penetration and accumulation within hair follicles, thereby augmenting their efficacy in targeted drug delivery to specific regions of the skin over an extended period [35]. The PDI was determined to have a value of 0.254 (Figure 2). The PDI is an indicator of the variability in the size distribution of nanocarriers within a formulation [36].

#### 2.3.2. Zeta Potential

**Figure 3:**
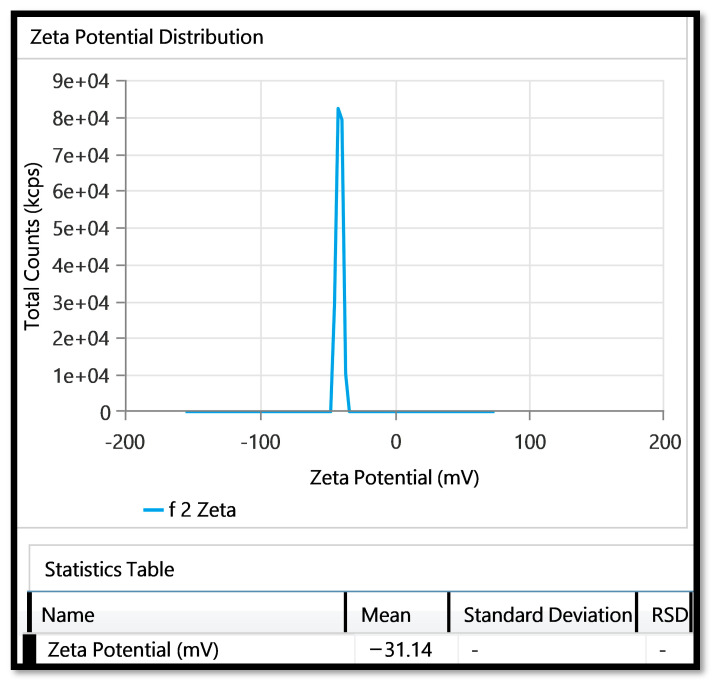
Zeta potential of Opt-HSD-loaded TE formulation.2.3.3. Determination of % Entrapment Efficiency and Drug Loading.

The zeta potential value of the optimized formulation was measured to be −31.14 mV, as depicted in Figure 3. The measurement of the zeta potential provides valuable insights into the stability of the formulation.

It was discovered that the optimized formulation’s entrapment efficiency and drug loading were 89.51% and 9.94%, respectively. The solubilization of hydrophobic drugs can be facilitated by the edge activators utilized in transethosomal formulations, hence improving the formulations’ drug entrapment efficiency as well as drug loading [37].

#### 2.3.3. TEM

**Figure 4:**
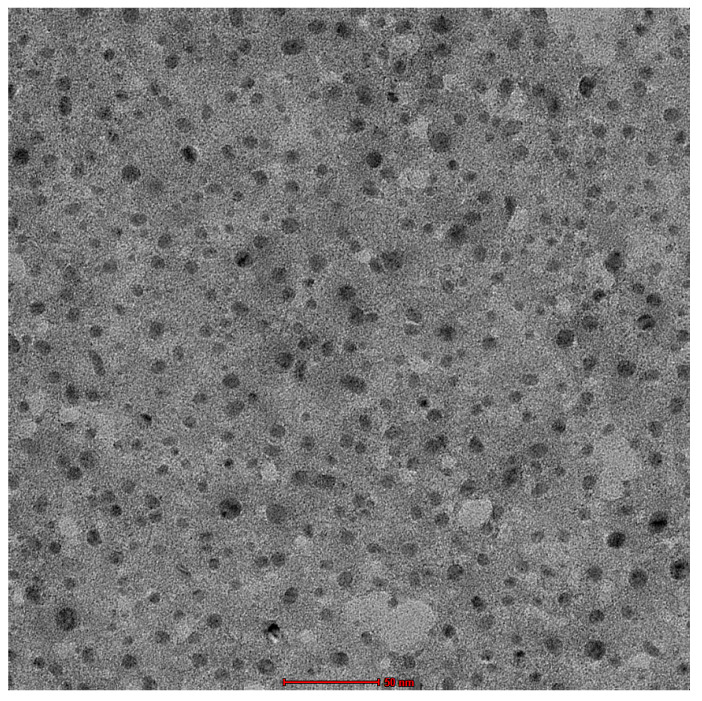
TEM image of Opt-HSD-loaded TE formulation.

The optimized HSD-TE formulation was analyzed using transmission electron microscopy (TEM) at a magnification of 100 kV. The results showed that the vesicles that were formed were spherical in shape (Figure 4). The observed differences in particle size measurements between TEM and DLS are the different measuring principles employed by the two methodologies. Particle interactions and aggregation can have an impact on the average hydrodynamic diameter reported by DLS, whereas TEM offers direct, high-resolution views of individual particles. The variances in reported particle sizes can be explained by these inherent differences in approach.

### 2.4. In Vitro Drug Release 

**Figure 5:**
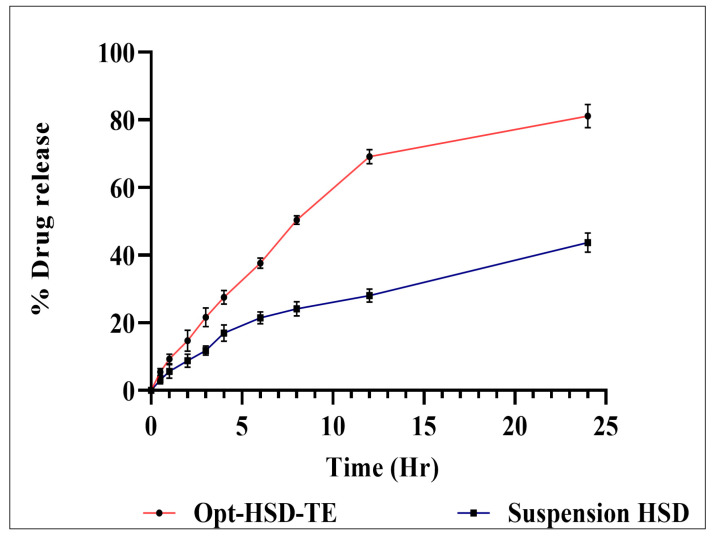
Comparative in vitro drug release of Opt-HSD-loaded TE and HSD suspension.

The Opt-HSD-TE formulation demonstrated an 81.124 ± 3.45% release of HSD via a dialysis membrane, compared to a 43.74 ± 2.89% in vitro HSD release from HSD suspension via a dialysis membrane (Figure 5). A considerable HSD release was accomplished at each interval. The HSD-TE formulation showed a delayed drug release when compared to HSD suspension. Because HSD can diffuse slowly and must cross the skin’s lipid bilayer, transethosomes can restrict drug release. The graph illustrates a quick release of HSD within the initial four-hour period, followed by a more gradual release over the subsequent twenty-four hours. The efficacy of treatment can be improved by engaging in this type of releasing behavior. In contrast to the rapid initial release, which contributes to achieving therapeutic concentrations, the extended slow release enhances therapeutic efficacy. First-order, zero-order, Korsmeyer–Peppas, and Higuchi mathematical kinetics models were employed to fit data from an in vitro drug release; these models gave the highest R^2^ value, which is shown in Table 3. As a result, it can be stated that the Higuchi model is the best-fit model, and a Korsmeyer–Peppas diffusion mechanism (non-Fickian diffusion) is involved in the release of HSD from the HSD-TE formulation. This is because the edge activator gives the vesicles elastic properties by disrupting the lipid bilayers without affecting the vesicle’s integrity. Additionally, ethanol works synergistically to increase flexibility and reduce vesicle particle size, making it easier for vesicles to pass through the dialysis membrane [38].

### 2.5. Preparation and Characterization of Gel

The transethosome gel containing HSD was effectively formulated. The physical properties of the transethosome gel loaded with HSD were assessed, and the gel was yellowish in color and translucent appearance. The HSD-TE gel that was optimized exhibited visual appeal and consistency, as there were no discernible abrasive particles observed throughout the entirety of the process. The gel that was formulated possesses a pH value of 6.19 ± 0.24, indicating that it falls within a safe range that is appropriate for the skin’s application of the gel [39]. The drug content in the optimized gel was found to be 85.19 ± 2.14. The convenience of the gel’s application by the patient to the affected area is a significant factor to be considered. The spreadability of the gel formulation, specifically the HSD-TE Gel, was determined to be 26.98 ± 1.98 g.cm/s. The high spreadability of gel indicates its improved ability to be applied onto a surface, while extrudability ensures smooth and consistent gel flow from its container.

#### Texture Analysis

**Figure 6:**
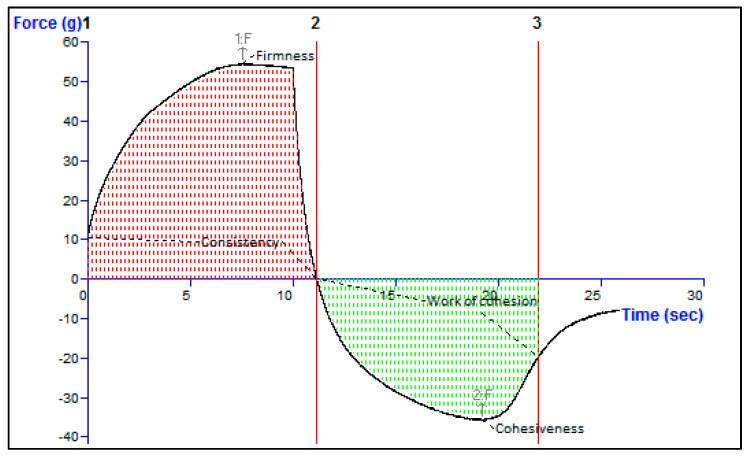
Texture analysis of Opt-HSD-loaded TE gel.

Texture analyses serve the dual purpose of facilitating in-process controls and providing insights into stability (Figure 6). The cohesiveness, consistency, firmness, and work of cohesion were found to be −35.96, 479.56 gm·s, 54.66 gm, and −294.04 g·s, respectively. The pertinent variables encompass consistency, firmness, cohesiveness, and work of cohesion. The term “Firmness” refers to the formulation’s ability to be applied to the skin. The cohesiveness of a gel relates to its ability to recover its structural network and resist deformation after application. Work of cohesion refers to the capacity to adhere to the skin [40]. 

### 2.6. Ex Vivo Skin Permeation Study

**Figure 7:**
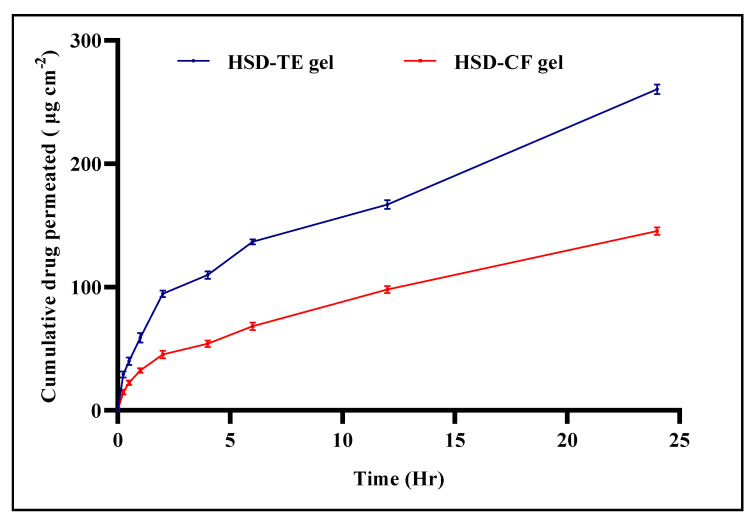
Comparative ex vivo permeability via skin of Opt-HSD-TE gel and HSD-CF gel.

The cumulative amount of drug permeated from Opt-HSD-TE gel was 260.49 ± 0.0187 μg cm^−2^, and from HSD-CF gel, the amount of cumulative drug permeated was 145.53 ± 0.0076 μg cm^−2^ via an excised rat skin (Figure 7). It was discovered that the steady-state flux for the HSD-TE gel and HSD-CF gel were 9.62 and 5.4851 μg cm^−2^ h^−1^, respectively. 

The increased deposition and permeation of HSD on the skin can be attributed to the characteristics and composition of the nanosized TE vesicles, which changed the arrangement of corneocytes and widened intercorneocyte openings via the skin’s hydration. This increased deposition of nanosized drug carriers in the epidermis and dermis region of the skin was the main cause of the improvement. They possess the ability to infiltrate the stratum corneum and minuscule skin pores. The improved delivery via the skin of these nanovesicles can be attributed to the inclusion of ethanol and an edge activator as permeation enhancers. In addition, it is possible that the inclusion of ethanol in phospholipid bilayer vesicles could enhance their flexibility, thereby allowing for modifications to the structure of the stratum corneum [41]. This, in turn, may facilitate deeper penetration into the skin layer and potentially enhance the permeation of drugs. The results of this study suggest that TE nanovesicles hold the potential for enhancing drug permeation into the deeper layers of rat skin in comparison to the unencapsulated drug [42].

### 2.7. CLSM

**Figure 8:**
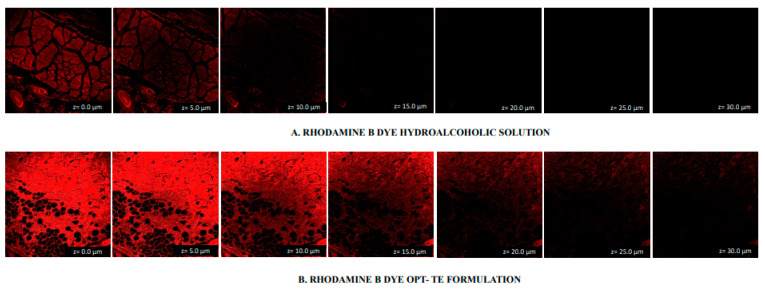
Penetration exhibited by CLSM images of (**A**) standard solution and (**B**) TE gel formulation on the rodent rat skin.

The utilization of confocal microscopy was employed to assess the depth of penetration achieved by both the rhodamine-loaded transethosome gel formulation and the standard solution. The findings of the study indicate that the standard solution exhibited penetration, as evidenced by the detection of a significant fluorescence of dye extending to a depth of 10.0 µm. Following this, the fluorescence intensity gradually decreased and eventually vanished (Figure 8A). In the case of the transethosome gel, it was found that the penetration was absorbed into the deeper layer of the skin, as shown by the existence of transcendent fluorescence that reached an extent of 25.0 µm (Figure 8B). 

### 2.8. Dermatokinetic

**Figure 9:**
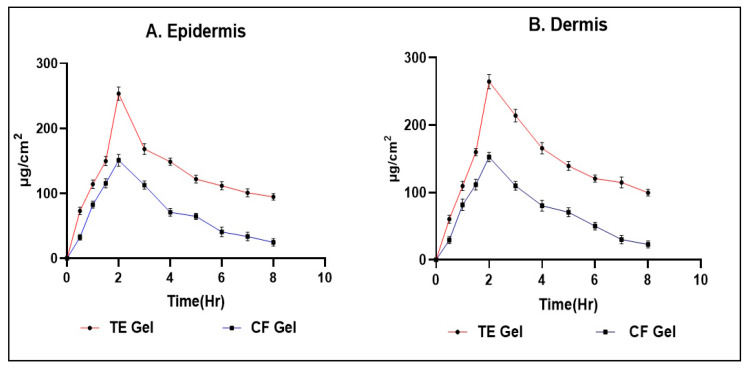
A comparison of the drug concentration (HSD) observed in the (**A**) epidermis and (**B**) dermis.

According to the study’s findings, the transethosome gel had higher drug concentrations than the standard formulation at all time periods in both the epidermal and dermal layers of the skin, as shown in Figure 9A,B. According to Table 4’s findings from the analysis, the transethosome gel showed greater Cskin-max and AUC0–8h values than a standard formulation in the epidermal and dermal layers of the skin. The study’s findings showed that the nanovesicle penetrated the skin’s barriers successfully and accumulated in the intended area at a sufficient concentration [43].

### 2.9. Antioxidant Activity

**Figure 10:**
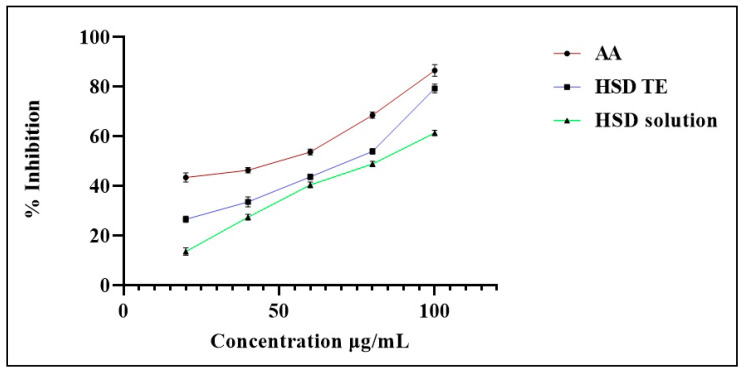
Antioxidant activity of HSD-TE in comparison with AA and free HSD.

Hesperidin has been shown to have significant antioxidant capacity, with tests demonstrating the agent’s efficacy in scavenging free radicals and preventing lipid peroxidation. Both the free HSD and the optimized HSD-TE formulation were tested for their antioxidant capacities and compared to a standard ascorbic acid solution. Antioxidant activity in ascorbic acid solution, free HSD, and optimized HSD-TE formulation was found to be 86.43 ± 2.37%, 61.30 ± 1.09%, and 79.20 ± 1.77%, respectively (Figure 10). The enhanced antioxidant action of Hesperidin-loaded transethosomes has been found to contribute to the elimination of radicals. The formulation exhibits the capability to enhance the skin’s inherent antioxidant capacity and assist in the mitigation of reactive oxygen species (ROS) and antioxidant activity shown to confer resistance against bacterial growth [44,45]. The IC_50_ values of AA, free HSD, and HSD-TE were found to be 42.01 μg/mL, 80.06 μg/mL, and 64.17 μg/mL, respectively. 

### 2.10. Antibacterial Activity

Hesperidin was examined for its antibacterial effects on two strains of bacteria, one Gram-negative (*E. coli*) and one Gram-positive (*S. aureus*). The bacteria were tested for susceptibility using the disc diffusion method. Table 5 displays the bactericidal properties of HSD, both free and encapsulated into the transethosome. HSD was found to be especially useful in preventing the spread of bacteria, a primary cause of many different skin conditions. The transethosome loaded with HSD was shown to be the most effective at reducing the growth of harmful bacterial strains (*S. aureus* and *E. coli*) at a dose of 30 µg/mL. 

#### 2.10.1. Minimum Inhibitory Concentration 

**Figure 11:**
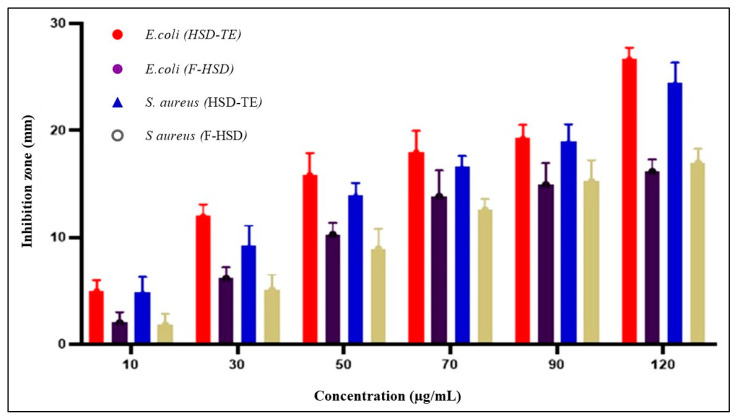
Antibacterial activity of free HSD and HSD-TE formulation (*p* < 0.05). The values are expressed as Mean ± SD (*n* = 3).

The antibacterial properties of HSD-TE exhibited an enhancement as the concentration of HSD-TE was elevated in comparison to the non-encapsulated HSD. The results of the antibacterial sensitivity analysis indicate that the E. coli and S. aureus strains exhibited the largest zone of inhibition, measuring 26.67 ± 1.09 mm and 24 ± 1.84 mm, respectively, when exposed to a concentration of 120 µg/mL of HSD-TE. These findings suggest that HSD-TE possesses a wide-ranging antibiotic efficacy, capable of combating both Gram-positive and Gram-negative bacterial species, whereas at 120 µg/mL, the free HSD showed 16.19 ± 1.09 mm and 17.36 ± 1.29 mm for *E. coli* and *S. aureus*, respectively (Figure 11). The zone of inhibition demonstrates that HSD-TE has significantly higher (*p* < 0.0001) antibacterial activity against free HSD. It shows a concentration dependent antibacterial activity.

#### 2.10.2. Minimum Bactericidal Concentration

The MBC can be discovered in the area where the MIC for the bacteria strain with the highest resistance matches the inhibitory zone. The MBC of free HSD and HSD-TE against *E. coli* and *S. aureus* was found to be 10 µg/mL.

### 2.11. Stability Studies

The results of assessments on short-term accelerated stability were evaluated for six months. The zeta potential, phase separation, PDI, clarity, homogeneity, pH, spreadability, and extrudability of the HSD-TE formulation and HSD-TE gel did not vary significantly during the experiment (Table 6 and Table 7). Thus, this research lends credence to the notion that nanovesicles can tolerate long-term storage.

## 3. Conclusions

Transethosomes were successfully used to encapsulate hesperidin, which is regarded to be a potential drug delivery method. The Box–Behnken design strategy was used to create several transethosomal formulations. Based on the values of a few examined criteria, the best formula, which contained phospholipid 90G (80 mg), ethanol (35%), and sodium cholate (10 mg), was selected, i.e., formulation batch 02. For better application, the optimized transethosome formulation was added to a Carbopol gel. In addition to having suitable physical properties for topical application, the transethosomal gel formulation also had efficient percutaneous absorption through the skin. The in vitro, ex vivo, dermatokinetic, and CLSM studies revealed the release, permeability, significant drug concentration, and penetration into the deeper layers of the rodent rat skin. These results suggest that transethosomes could hold promising potential for in vivo applications in anti-bacterial treatments. This formulation demonstrates the ability to effectively decrease the presence of free radicals and reactive oxygen stress, hence exhibiting potential antibacterial properties. Compared to free hesperidin, the antibacterial activity and MIC of transethosome loaded with hesperidin were found to be more potent. The TE formulation was developed as a drug carrier for topical hesperidin administration because ethosomes are nanocarriers that can fuse into microbial cells while boosting the potency of antibacterial drugs. Furthermore, the experimental findings validate the efficacy of the optimized transethosome formulation in facilitating the topical administration of Hesperidin, hence enhancing the potential for more effective treatment of antibacterial conditions. Nevertheless, the extent of skin permeation of hesperidin may differ based on the specific formulation and unique attributes of the skin. Although hesperidin is generally considered safe, the safety profile of transethosomes containing HSD for topical administration has not been thoroughly established, as in vivo studies were not conducted in the current study. Further investigation is necessary to ascertain their potential toxicity and adverse effects in a suitable clinical model; more research is required.

## 4. Materials and Methods

### 4.1. Materials

Hesperidin was purchased from Sigma-Aldrich (St. Louis, MO, USA). Phospholipid 90G was procured from Lipoid, Germany. Carbopol 934P, Polyethylene glycol, and Triethanolamine were procured from Sigma-Aldrich (St. Louis, MO, USA). Rhodamine B, Ascorbic acid, 1,1diphenyl-1-picrylhydrazy (DPPH) were procured from SD Fine Chemicals, Mumbai, India. Rodent rat skin was utilized from Etsy Pvt Ltd. India. All the other reagents and chemicals used in the experiments were of analytical grade.

### 4.2. Development of Hesperidin-Loaded Transethosomes

A predetermined amount of Lipoid S 100, sodium cholate, and hesperidin were dissolved in a mixture of methanol and chloroform (in a volumetric ratio of 1:2) within a round bottom flask. Subsequently, the organic solvents were eliminated via vacuum evaporation with reduced pressure utilizing a rotary evaporator (Model Eyela N-1000 series, Tokyo Rikakikai Co., Ltd., Bunkyo-ku, Japan) until a thin layer of lipid mixture was formed on the inner surfaces of the round bottom flask. The thin lipid film that was deposited underwent a vacuum environment to ensure the complete removal of any residual solvents. The lipid film that had been dried and deposited was rehydrated by exposure to a hydroethanolic solution. The prepared dispersions were sonicated using an ultrasonicator (UP100H, Hielscher Ultrasonics GmbH, Berlin, Germany) for 2 min. The developed formulation was optimized and characterized for various parameters [23].

### 4.3. Optimization of Transethosomes

The optimization process of the formulation containing hesperidin-loaded transethosomes (HSD-TE) was conducted utilizing the Box–Behnken Design (BBD) approach, employing the Design Expert^®^ software version 13. As shown in Table 8, three different components were chosen as independent variables: Phospholipid 90G (mg), ethanol (%) and sodium cholate (mg) (A, B, C), while particle size (nm) (Y_1_), PDI (Y_2_), and percentage entrapment efficiency (EE%) (Y_3_) were designated as response variables. A series of 17 experimental runs have been carried out utilizing a 3-level, 3-factor Box–Behnken design (BBD), as shown in Table 1. Using the ANOVA feature offered by the software, the statistical validity of the polynomial equations produced by Design Expert^®^ was validated.

### 4.4. Characterization of HSD-Loaded Transethosomes

#### 4.4.1. Particle Size and Polydispersity Index

The average particle size and polydispersity index (PDI) of the transethosome that were prepared were determined using a Zeta sizer instrument (Malvern Instruments, Worcestershire, UK). The dispersion of prepared transethosome (100 µL) was diluted to a volume of 2 mL using Milli-Q water. The diluted sample was then transferred into disposable polystyrene cuvettes and subsequently inserted into the Zeta sizer and measured [46].

#### 4.4.2. Zeta Potential Measurement

To evaluate the charge on the surface, the zeta potential was examined, and it was determined that a stable condition was achieved when the value fell within the range of less than −30 and greater than +30. The electrostatic potential, also known as the zeta potential, exhibits a direct relationship with the surface charge of nanovesicles. Nanovesicles exhibiting a zeta potential within the range of +10 mV to −10 mV is commonly regarded as possessing a neutral charge. Before conducting the investigation, a 100-fold dilution of the samples was achieved by employing milli-Q water. Additionally, impurities were eliminated from the samples by utilizing 0.45 µm membrane filters [47].

#### 4.4.3. Measurement of Entrapment Efficiency and Drug Loading Percentage

The entrapment efficiency and drug loading percentage are important parameters in drug delivery systems. The measure of entrapment efficiency pertains to the quantity of drug trapped or encapsulated within the nanovesicles. Drug loading refers to the quantification of the drug content within nanovesicles relative to their weight. The HSD-loaded transethosome dispersion (1 mL) was subjected to ultracentrifugation (Cooling Centrifuge, C24, REMI Ins. Ltd., Mumbai, India) using an Eppendorf centrifuge at a speed of 15,000 revolutions per minute for a duration of 30 min at a temperature of 4 degrees Celsius in order to facilitate the sedimentation of the transethosome. The diluted supernatant was subjected to analysis using UV spectroscopy in conjunction with methanol [48]. The entrapment efficiency and drug loading were determined using the equations below:

$$\%Entrapment\;efficiency=\frac{Total\;amount\;of\;HSD\;entrapped}{Total\;amount\;of\;HSD\;added}\times 100$$

$$\%Drug\;loading=\frac{Amount\;of\;drug\;entrapped}{Amount\;of\;drug\;and\;lipid}\times 100$$

#### 4.4.4. Transmission Electron Microscopy (TEM)

The vesicle morphology of the optimized formulation of transethosomes was assessed via the utilization of TEM using the Joel JEM 1230 instrument from Tokyo, Japan. To create a thin film on the grid, the sample was positioned on a copper grid coated with carbon. A solution of phosphotungstic acid (PTA 1% *v*/*v*) was applied to the grid. The surplus solvent was removed using filter paper and subsequently analyzed using transmission electron microscopy (TEM) at a voltage of 100 kV [49].

### 4.5. In Vitro Drug Release and Kinetic Models

The in vitro drug release of the optimized HSD-TE formulation was analyzed using a dialysis bag with a molecular weight cutoff of 12,400 Da, obtained from Sigma-Aldrich Co. in St. Louis, MO, USA. The 2 mL of HSD-TE formulation solution and HSD dispersion were placed into the dialysis bags and sealed at both ends. During the experiment, the dialysis bags were immersed in a beaker containing 100 mL of phosphate-buffered saline (pH 7.4). The beaker was subjected to magnetic stirring at a rate of 100 revolutions per minute and maintained at a temperature of 37 ± 0.2 °C. The samples were taken and subjected to analysis at various time intervals (0, 0.5, 1, 2, 3, 4, 6, 8, 12, and 24 h) while ensuring sink conditions. The analysis involved spectrophotometric measurement of the drug concentration at a wavelength of 280 nm [50].

#### Release Kinetic Models

Various release kinetics were employed to investigate the mechanism of drug release. The release rate data were subjected to fitting procedures using various release kinetics models, including zero order, first order, Higuchi model, and Korsemeyer–Peppas model. The model with the highest R^2^ value was chosen as the best fit [51].

### 4.6. Preparation of Carbopol-Based HSD-Loaded Transethosome Gel

The HSD-TE underwent gel conversion via the incorporation of the sample into carbopol 934P. The carbopol 934P was used as a gelling agent, and it was dispersed in double distilled water. The mixture was allowed to remain undisturbed for an extended period, specifically overnight, to ensure thorough expansion and solidification. Subsequently, the optimized HSD-loaded transethosome was introduced into the carbopol mixture under constant stirring. Subsequently, PEG 400 (5%) as a humectant and plasticizer and triethanolamine (0.5%) were incorporated and homogenized to neutralize; later, a clear gel was obtained [52].

### 4.7. Characterization of HSD-Loaded TE Gel

#### 4.7.1. Physical Inspection

The evaluation of gel formulation homogeneity and color was typically conducted via visual examination, whereby the formulated gel was applied onto a transparent material such as glass, followed by inspection [53].

#### 4.7.2. Texture Analysis

Backward extrusion measurements were conducted using a Texture Analyzer TA. XT Plus (Stable Micro Systems Ltd. Surrey, UK). A circular object with a diameter of 40 mm was inserted into a carbopol-based HSD-loaded TE gel substance weighing 60 g. With a rate of 4 mm/s, the disc was inserted into the hydrogel and then removed. The measurement of the hardness of the gel was conducted, and various texture properties, including cohesiveness and adhesiveness, were subsequently computed. The highest force reached during the disc’s downward motion served as the hardness indicator. Cohesiveness refers to the level of exertion required to compact the disc within the gel that has been created, whereas adhesiveness is the effort necessary to move the disc upward. Adhesiveness measures how well the formulation adheres to the disc [54].

#### 4.7.3. Determination of Spreadability and Extrudability

Spreadability is quantified by measuring the duration in seconds. It takes two slides to detach from a gel surface when subjected to a specific load. A shorter separation time indicates a higher level of spreadability. The spreadability of each formulation was replicated three times in order to determine its consistency [55].

S = M × L/T

where M represents the weight attached to the upper slide, L represents the length of the glass slides, and T represents the duration required for the process of separating the slides.

The collapsible tubes were filled with the gel formulations within the container. The extrudability of the formulation was assessed by measuring the weight in grams needed to extrude a gel ribbon with a thickness of 0.5 cm within a time frame of 10 s.

#### 4.7.4. Measurement of pH 

The gel’s pH was measured using a digital pH meter (MW802, Milwaukee Instruments, Szeged, Hungary). One gram of gel was dissolved in 100 mL of distilled water, and the mixture was stirred until it became a homogenous solution. Subsequently, the electrode was immersed into the gel formulation until a stable measurement was achieved. The measurements were conducted in triplicate.

### 4.8. Determination of Drug Content

A predetermined amount of gels (10 mg) was mixed with 10 mL of methanol using a vortex mixer, followed by filtration through Whatman filter paper. A 1 mL portion of the filtrate was diluted with 4 mL of methanol. The resulting mixture was then filtered, and the absorbance was subsequently measured at a wavelength of 280 nm using a UV spectrophotometer (Shimadzu UV-1800 Double beam Spectrophotometer, Japan) [56].

### 4.9. Ex Vivo Skin Permeation Study

The researchers employed rat skin as an imitation to replicate the characteristics of human skin [46]. The ex vivo permeation experiment was conducted using Franz diffusion cells with a diffusional area of 0.758 cm^2^. The rat skin was positioned between the receptor and donor compartments of the Franz cell. Subsequently, a volume of 10 mL of phosphate-buffered saline (PBS) with a pH of 7.4 was transferred to the receptor compartment. The diffusion cell was then subjected to magnetic stirring maintained at a temperature of 37 ± 1 °C. The optimized HSD-TE gel formulation and HSD dispersion gel (conventional formulation) were introduced into the donor compartment. Subsequently, 1 mL samples were withdrawn from the receptor medium at various time intervals ranging from 0.5 to 24 h. Following the withdrawal of each sample, an equal volume of a prepared buffered solution was introduced to maintain sink conditions. The drug concentrations were then measured using UV spectrophotometry at a wavelength of 280 nm. To prevent any potential interference, every sample was contrasted with a control sample devoid of the drug. The calculation of the permeation flux (Jmax) at 24 h was performed using the equations provided below [57].

$$Jmax=\frac{Amount\;of\;drug\;permeated}{Time\times area\;of\;the\;membrane}$$

### 4.10. Confocal Laser Scanning Microscopy (CLSM)

The study utilized a Confocal Laser Scanning Microscopy 410 (Zeiss, Heidelberg, Germany) to evaluate the penetration depth and visualize the permeation into the skin from both Rhodamine B dye-loaded transethosome gel and standard hydroalcoholic rhodamine B solution formulations. The skin affixed to the Franz diffusion cell underwent treatment with a formulation and standard solution. After a duration of 8 h, the skin was removed and subsequently treated with ethanol to eliminate any residual traces of the formulation and solution that may have adhered to it. Furthermore, the stratum corneum was oriented vertically, and the slides were seen under a confocal microscope at a wavelength of 488 nm. The fluorescence emission from the argon laser and wavelengths longer than 560 nm were seen. Confocal laser scanning microscopy (CLSM) analysis of the slides was used to determine the fluorescence intensity within the various layers of the skin [58].

### 4.11. Dermatokinetic Study

The method of measuring the concentration of drugs in various skin layers is referred to as dermatokinetic. Using a Franz cell diffusion assembly, the experiment used skin samples that were removed from rat skin. Following the instructions provided in the ex vivo skin permeation investigation, the skin membrane was prepared. A gel was administered to the donor compartment that included HSD-TE and HSD-conventional gel (HSD-CF gel), with 1.0 g of gel containing 1 mg HSD. The experiment was carried out in accordance with the steps described in the ex vivo skin permeation investigation. In contrast, the complete skin from the Franz cell was removed for this study at the predetermined sampling intervals (0, 0.5, 1, 1.5, 2, 3, 4, 5, 6, 7, and 8 h). To remove any leftover formulation that might have stuck to the skin, the skin samples were put through washing with regular saline solution. The skin sample was immersed in water heated to a temperature of 60 °C for a duration of 2–3 min. Subsequently, the layers of the epidermis and dermis were carefully separated using forceps with fine and sharp points. The two layers were individually fragmented into small fragments and subjected to maceration in 10 mL of methanol for a duration of 24 h to facilitate the extraction of drugs. Subsequently, the methanolic solution underwent filtration using a filter with a pore size of 0.45 µm. The concentration of HSD in the solution was measured using spectrophotometry at a wavelength of 280 nm. Separate graphs for HSD concentration per cm square of skin vs. time were plotted for the epidermis and dermis. The dermatokinetic parameters, including Tskin max, Cskin max, AUC0–8h, and Ke, were determined using PK solver software version 3.0 [59].

### 4.12. Antioxidant Activity

The overproduction of free radicals and a buildup of reactive oxygen species (ROS) are the two characteristics that characterize oxidative stress [60]. ROS is one of the contributions to bacterial diseases [61]. Antioxidants are frequently utilized to inhibit the free radicals’ formation. Hesperidin possesses the capacity to act as antioxidants [62]. The ability of the HSD to neutralize the stable nitrogen-centered free radical 2,2-diphenyl-1-picrylhydrazyl (DPPH). This study compared the antioxidant activity of HSD-TE to that of the gold standard ascorbic acid (AA) solution. A 25 μM concentration of DPPH was achieved by dissolving it in methanol. All three samples were prepared in methanol at different concentrations (20–100 μg/mL), and one milliliter of the DPPH methanolic solution was added to each sample to dilute it. The mix was then stored at room temperature for a duration of 30 min while being kept in darkness. Subsequently, the measurement of absorbance was conducted at 517 nm, with the blank solution consisting of methanol serving as the reference. The degree of violet coloration reduction in a methanolic solution of DPPH is contingent upon the inherent antioxidant activity or radical scavenging activity, as well as the concentration of the sample. Antioxidant compounds possess the ability to counteract the DPPH radical via two distinct mechanisms: either radical quenching aided by hydrogen atom donation or direct reduction aided by electron donation. Each sample’s ability to scavenge DPPH radicals was evaluated as a percentage of antioxidant activity using the formula below [63].

$$Scavenging\%=\frac{Absorbance\;of\;blank-absorbance\;of\;sample}{Absorbance\;of\;blank}\times 100$$

### 4.13. Determination of Anti-Bacterial Activity Using Well Diffusion Method

The agar well diffusion technique was used to examine the HSD-loaded transethosome’s antibacterial efficacy. The bacteria were subcultured in a broth medium and incubated at 37 °C for 24 h to produce a uniform microbial growth plate. The next step was to spread overnight cultures across the agar plates. The two types of bacteria were *E. coli* (Gram-negative) and *S. aureus* (Gram-positive), and Gentamicin was used as a standard for assessing antibacterial efficacy. The Petri dishes were then kept in an incubator at 37 °C for a further 24 h. To evaluate the antibacterial activity of the manufactured HSD-loaded transethosome, the diameter of the inhibition zone was measured and compared to the control group [64].

#### 4.13.1. Determination of Minimum Inhibitory Concentration (MIC)

The minimum inhibitory concentration (MIC) was determined as the concentration of antibacterial drugs that effectively suppressed microbial growth after a 24 h incubation period. The technique of disc diffusion was employed to assess free HSD (F-HSD) and HSD-loaded TE. The objective was to ascertain the minimum inhibitory concentration (MIC) of these forms and evaluate their effectiveness in reducing bacterial strains. The experiment involved preparing various concentrations (10 µg/mL to 500 µg/mL) of F-HSD and HSD-TE. The Mueller-Hinton agar medium was employed to inoculate sterile Petri dishes with potentially pathogenic bacterial cultures. Different concentrations of F-HSD and HSD-TE were acquired and subsequently applied onto filter-paper discs. These discs were subsequently positioned on the surface of Mueller-Hinton agar plates. Following a refrigeration period of 2 h at 5 °C, the plates were subsequently subjected to incubation at a temperature of 35 °C for a duration of 24 h. The measurement of inhibition zones was conducted utilizing Vernier calipers, and subsequently, a comparison was made between these measurements [32].

#### 4.13.2. Determination of Minimum Bactericidal Concentration

Following the Minimum Inhibitory Concentration (MIC) study, further investigation was conducted by subculturing each well that exhibited no visible growth onto an agar medium to determine the Minimum Bactericidal Concentration (MBC) value. Following a 24 h incubation period at a temperature of 37 °C, the bacterial colony present on the agar plate was enumerated. The minimum concentration at which the sample effectively eradicated all bacteria is referred to as the minimum bactericidal concentration (MBC). The MBC was determined by identifying the concentration of the sample that resulted in a 99.9% reduction in the viability of the initial bacterial inoculum [65].

### 4.14. Stability Studies

The transethosome optimized formulation and gel formulation were stored for three months at 40 ± 2 °C/75 ± 5% RH. The formulation was monitored by taking samples at 0, 30, 60, and 180 days. Phase separation, vesicle size, polydispersity index, and zeta potential were chosen as the parameters for the stability assessment. The appearance, homogeneity, spreadability, extrudability, and pH of the gel were also measured.

### 4.15. Statistical Analysis

The statistical analysis of the data was verified using GraphPad Prism (v 5.0, GraphPad Software Inc., San Diego, CA, USA), and one-way analysis of variance (ANOVA) procedures were used. A mean and standard deviation (*n* = 3) were used to summarize the results.

## Figures and Tables

**Table 1:** The response values in the BBD were observed for optimizing the formulations of HSD-loaded transethosomes using the Design Expert Software version 13.0.

Independent Factors				Responses		
Run	A (Phospholipid) (mg)	B (Ethanol) (%)	C (Sodium Cholate) (mg)	Y_1_ (Vesicle Size) (nm)	Y_2_ (PDI)	Y_3_ (%EE)
1	60	35	15	204.34	0.291	56.19
2	80	35	10	178.98	0.254	89.51
3	80	55	15	181.28	0.288	81.58
4	80	35	10	177.56	0.261	88.14
5	80	35	10	178.14	0.271	87.46
6	80	55	5	181.77	0.249	80.13
7	100	35	15	210.56	0.319	76.77
8	60	35	5	202.14	0.315	60.23
9	100	15	10	204.44	0.31	79.58
10	100	35	5	211.98	0.301	78.66
11	80	35	10	178.99	0.277	85.23
12	60	55	10	198.18	0.298	66.89
13	80	15	5	185.1	0.299	78.98
14	60	15	10	196.55	0.294	54.89
15	80	35	10	178.98	0.27	86.85
16	80	15	15	184.24	0.244	79.34
17	100	55	10	203.88	0.304	80.98

**Table 2:** Statistical parameters for the BBD design are listed below.

Responses	Analysis	R^2^	R^2^ (Adjusted)	R^2^ (Predicted)	Std	CV (%)	Model	*p*-Value
Vesicle size (nm)	Polynomial	0.9962	0.9913	0.9489	1.18	0.6179	Quadratic	<0.0001
PDI	Polynomial	0.9600	0.9085	0.9005	0.0071	2.70	Quadratic	0.0004
% EE	Polynomial	0.9844	0.9644	0.8255	2.07	2.68	Quadratic	<0.0001

**Table 3:** Different kinetics models of drug release mechanism.

Kinetics Release	Korsmeyer–Peppas	Higuchi	Zero Order	First Order	Korsmeyer–Peppas	Higuchi	Zero Order	First Order
	R^2^	*n*	R^2^	K	R^2^	*n*	R^2^	K
	0.9861	0.7455	0.9668	0.0258	0.8778	0.0006	0.9632	0.0005

**Table 4:** Dermatokinetic parameters of HSD-TE gel compared to HSD-CF gel in epidermis and dermis layer of skin.

Parameters of Dermatokinetic	HSD-TE Gel		HSD-CF-Gel	
	Epidermis	Dermis	Epidermis	Dermis
T_skin max_ (h)	2	2	2	2
C_skin max_ (μg/cm^2^)	295.58 ± 3.09	264.59 ± 1.99	150.56 ± 2.56	152.44 ± 0.99
AUC_0–8_ (μg/cm^2^ h)	1087.58 ± 2.99	1167.12 ± 2.11	561.12 ± 1.24	579.14 ± 0.56
Ke (h^−1^)	0.0371 ± 0.11	0.01143 ± 0.78	0.01142 ± 0.54	0.1361 ± 0.41

**Table 5:** Antimicrobial screening test of free HSD and HSD-loaded TE (30 µg/mL) against Gram-positive and negative bacteria.

Microorganism	Hesperidin		Gentamycin (20 µg/mL)
	Free HSD (30 µg/mL)	HSD-Loaded TE (30 µg/mL)	
*S. aureus*	14.27 ± 0.84	18.11 ± 0.54	20.47 ± 0.96
*E. coli*	12.19 ± 0.69	17.24 ± 0.78	18.94 ± 0.46

**Table 6:** Assessment of the accelerated short-term stability of the transethosome formulation loaded with HSD.

Parameters of Evaluation	Initial	30 Days		90 Days		180 Days	
		4 ± 2 °C	25 ± 2 °C/ 60 ± 5%RH	4 ± 2 °C	25 ± 2 °C/ 60 ± 5%RH	4 ± 2 °C	25 ± 2 °C/ 60 ± 5%RH
Appearance	***	***	**	***	***	**	**
Separation of phase	×	×	×	×	×	×	×
Vesicle size (nm)	164.96	165.14	167.29	171.37	173.44	176.69	178.78
PDI	0.254	0.254	0.261	0.289	0.299	0.305	0.307
Zeta potential (mV)	−31.26	−31.30	−32.04	−32.48	−32.99	−33.14	−33.55

** Satisfactory, *** Excellent, ^x^ No.

**Table 7:** Evaluation of the HSD-loaded transethosome gel formulation’s stability for short-term accelerated.

Parameters of Evaluation	Initial	01 Month		03 Months		06 Months	
		4 ± 2 °C	25 ± 2 °C/60 ± 5% RH	4 ± 2 °C	25 ± 2 °C/ 60 ± 5% RH	4 ± 2 °C	25 ± 2 °C/60 ± 5% RH
Color	Yellowish	Yellowish	Yellowish	Yellowish	Yellowish	Yellowish	Yellowish
Clarity	***	***	***	***	***	***	**
pH	6.19	6.21	6.99	7.01	6.88	6.94	6.77
Homogeneity	***	***	**	***	**	***	*
Extrudability	***	***	**	***	**	***	**
Spreadability	***	***	**	***	***	***	**

* Good, ** Satisfactory, *** Excellent.

**Table 8:** Box–Behnken design (BBD) independent and dependent variables for the development and optimization of STCN-TL.

Variables	Used Levels		
	Minimum (−1)	Medium (0)	Maximum (+1)
**Independent variables**			
A = Phospholipid 90G (mg)	60	80	100
B = Ethanol (%)	15	35	55
C = Sodium cholate (mg)	05	10	15
**Dependent variables**			
Y_1_ = Vesicles size (nm)			
Y_2_ = PDI			
Y_3_ = EE %			

## Data Availability

The data will be available from the authors upon reasonable request.
